# The Specific Connexin 43–Inhibiting Peptide Gap26 Improved Alveolar Development of Neonatal Rats With Hyperoxia Exposure

**DOI:** 10.3389/fphar.2021.587267

**Published:** 2021-07-05

**Authors:** Cai Qing, Zhao Xinyi, Yu Xuefei, Xue Xindong, Fu Jianhua

**Affiliations:** Department of Pediatrics, Shengjing Hospital of China Medical University, Shenyang, China

**Keywords:** bronchopulmonary dysplasia, apoptosis, oxidative stress, intercellular communication, connexin 43, gap junction

## Abstract

Bronchopulmonary dysplasia (BPD) is a common devastating pulmonary complication in preterm infants. Alveolar maldevelopment is the crucial pathological change of BPD highly associated with oxidative stress–mediated excessive apoptosis. Cellular injury can be propagated and amplified by gap junction (GJ)–mediated intercellular communication. Connexin 43 (Cx43) is the most ubiquitous and critical GJ protein. Gap26 is a specific Cx43 mimic peptide, playing as a Cx43-GJ inhibitor. We hypothesized that Cx43-GJ was involved in alveolar maldevelopment of BPD *via* amplifying oxidative stress signaling and inducing excessive apoptosis. Neonatal Sprague Dawley rats were kept in either normoxia (21% O_2_) or hyperoxia (85% O_2_) continuously from postnatal day (PN) 1 to 14 in the presence or absence of Gap26. Moreover, RLE-6TN cells (type II alveolar epithelial cells of rats) were cultured *in vitro* under normoxia (21% O_2_) or hyperoxia (85% O_2_). RLE-6TN cells were treated by N-acetyl cysteine (NAC) (a kind of reactive oxygen species (ROS) scavenger) or Gap26. Morphological properties of lung tissue are detected. Markers associated with Cx43 expression, ROS production, the activity of the ASK1-JNK/p38 signaling pathway, and apoptotic level are detected *in vivo* and *in vitro*, respectively. *In vitro*, the ability of GJ-mediated intercellular communication was examined by dye-coupling assay. *In vitro*, our results demonstrated ROS increased Cx43 expression and GJ-mediated intercellular communication and Gap26 treatment decreased ROS production, inhibited ASK1-JNK/p38 signaling, and decreased apoptosis. *In vivo*, we found that hyperoxia exposure resulted in increased ROS production and Cx43 expression, activated ASK1-JNK/p38 signaling, and induced excessive apoptosis. However, Gap26 treatment reversed these changes, thus improving alveolar development in neonatal rats with hyperoxia exposure. In summary, oxidative stress increased Cx43 expression and Cx43-GJ–mediated intercellular communication. And Cx43-GJ–mediated intercellular communication amplified oxidative stress signaling, inducing excessive apoptosis *via* the ASK1-JNK/p38 signaling pathway. The specific connexin 43–inhibiting peptide Gap26 was a novel therapeutic strategy to improve the alveolar development of BPD.

## Introduction

Bronchopulmonary dysplasia (BPD) is the most common chronic lung disease in premature infants (Siffel et al., 2021), especially in those requiring oxygen supplementation or mechanical ventilation during treatment (Abman et al., 2017; Principi et al., 2018). A key histopathological feature of BPD is alveolar maldevelopment, which is characterized by fewer and larger alveoli (Kalikkot et al., 2017; Hwang and Rehan, 2018). Aberrant alveolarization results in pulmonary dysfunction in infants with BPD which lasts into adulthood (Lignelli et al., 2019). Besides, BPD is an independent risk factor for long-term neurodevelopmental impairment (Jensen and Schmidt, 2014). The overall incidence of BPD in infants born at <28 weeks of gestational age is estimated to be 48–68% (Stoll et al., 2010). The lower the gestational age and birth weight, the higher the incidence of BPD, with about 80% incidence in preterm infants with a gestational age of 22–24 weeks (Siffel et al., 2021). Thus, therapies that decrease BPD incidence would have a significant impact on morbidity, mortality, human costs, and healthcare expenditure (Savani, 2018).

Oxygen supplementation is a lifesaving therapeutic measure used for premature infants with pulmonary insufficiency. However, premature infants with immature antioxidant defense systems are more susceptible to oxidative stress (Kinsella et al., 2006). Oxidative stress–induced excessive apoptosis of alveolar epithelial cells is the crucial mechanism in alveolar maldevelopment of BPD (Das et al., 2004; De et al., 2008; Zhang et al., 2018). Apoptosis signal–regulated kinase 1 (ASK1)–mediated apoptosis is involved in the pathogenesis of several oxidative stress–related diseases such as brain ischemia (Zhang et al., 2003), ischemic heart disease (Watanabe et al., 2005), and Alzheimer’s disease (Kadowaki et al., 2005). Under oxidative stress conditions, ASK1 resulted in apoptosis by activating pro-apoptotic c-Jun NH2-terminal kinase (JNK) and p38 mitogen–activated protein kinase (MAPK) pathway (Tobiume et al., 2001). Previous studies indicated JNK and p38 MAPK pathway had been most frequently reported to have roles in the induction of apoptotic responses in hyperoxia lung injuries (Li et al., 2003; Romashko et al., 2003; Huang et al., 2009).

Intercellular communication is critical for the propagation and amplification of cellular injury. Gap junction (GJ) channels provide one of the most common forms of intercellular communication. GJ channels are formed by paired connexons, which are present on the membranes of adjacent cells. Each connexon is made up of six connexin (Cx) subunits. Cxs are transported from the endoplasmic reticulum *via* the Golgi apparatus and the *trans*-Golgi network to the cell membrane. GJ channels allow the exchange of ions, signaling molecules, and various metabolites between cells of most tissues, including reactive oxygen species (ROS), glutathione, calcium, trisphosphate, glutathione, cyclic guanosine monophosphate, and cyclic adenosine monophosphate (Goodenough and Paul, 2009). Of the 21 human Cx genes, connexin 43 (Cx43) is the most ubiquitous and critical GJ protein in many tissues (Sohl and Willecke, 2004). GJ channels under cellular stresses may also be hazardous by providing a channel to spread cytotoxicity to the adjacent cells and amplify cell injury (Lin et al., 1998; Perez et al., 2003; Spray et al., 2013). For example, Cx43 channels propagate radiation-induced DNA damage to non-irradiated brain microvascular endothelial cells (Hoorelbeke et al., 2020). In obesity, Cx43-mediated cell–cell coupling allows endoplasmic reticulum stress (ER stress) signals to disseminate between cells in the liver, leading to fatty liver disease and metabolic alterations (Tirosh et al., 2021). In a study of sepsis-induced intestinal injury, Cx43 channels regulate ROS generation and distribution between intestinal epithelial cells, thus regulating the activity of the JNK1/Sirt1/FoxO3a signaling pathway, resulting in the expression of pro-apoptotic Bim and Puma and sepsis-induced intestinal injury aggravation (Zou et al., 2019).

Previous studies have found that Cx43 is involved in a variety of lung diseases such as acute lung injury, asthma, and cystic fibrosis (Abraham et al., 1999; Parthasarathi et al., 2006; Sarieddine et al., 2009; Kandasamy et al., 2015). However, the role of Cx43 and Cx43-GJ in BPD is unclear. We hypothesized that Cx43-GJ was involved in alveolar maldevelopment of BPD *via* amplifying oxidative stress signaling and inducing excessive apoptosis. To confirm our hypothesis, we treated the neonatal rats in 21% O_2_ or 85% O_2_ with a specific Cx43 mimic peptide (Gap26). Gap26 is a kind of Cx43-GJ inhibitor, blocking Cx43-mediated cell–cell coupling. Moreover, RLE-6TN cells (type II alveolar epithelial cells of rats) were cultured *in vitro* in 21% O_2_ or 85% O_2_ and treated with N-acetyl cysteine (NAC) (a kind of ROS scavenger) or Gap26.

## Materials and Methods

### Animal Models and Treatment

All Sprague Dawley (SD) rats were purchased from the Laboratory Animal Center, Shengjing Hospital of China Medical University (Shenyang, China). The gestational age of the newborn rats was 21–23 days. According to our established procedure (Hou et al., 2015; Zhu et al., 2015; Zhang et al., 2018), neonatal rats were exposed to 21% O_2_ (normoxia) or 85% O_2_ (hyperoxia) from postnatal day (PN) 1 to 14. A total of one hundred eighty neonatal rats were randomly divided into six groups: normoxia group (*n* = 30); hyperoxia group (*n* = 30); normoxia and saline group (*n* = 30); hyperoxia and saline group (*n* = 30); normoxia and Gap26 group (*n* = 30); and hyperoxia and Gap26 group (*n* = 30). Some neonatal rats were treated with Gap26 (Apexbio, United States) *via* intraperitoneal injection at 50 μg/kg body weight once daily from PN1d to PN14d (Li et al., 2015), and some were treated with the same amount of sterile saline. The rat living environment was as follows: 12 h alternating light/dark cycle, and temperature was 25–26°C, with humidity at 60–70%. Each group was adjusted to 6–8 pups to minimize the effects of nutrition differences on lung development. Oxygen concentration in the oxygen chamber was continuously monitored using an oxygen meter. The concentration of CO_2_ was maintained at <0.5% by absorbing CO_2_ with soda lime. The mother rats in 85 and 21% O_2_ were exchanged once every 24 h to eliminate feeding differences and avoid oxygen poisoning. Cages were regularly opened for 30 min every day to replace the padding and provide clean drinking water and food. All animal procedures were approved by the Laboratory Animal Ethics Committee of Shengjing Hospital of China Medical University.

The rats were anesthetized by sevoflurane inhalation; then, the thoracic cavities were immediately opened, and lung tissues were dissected. Lung samples were lavaged at 18 cm H_2_O pressure with precooled phosphate-buffered saline (PBS) (4°C). The left lungs were fixed in 4% paraformaldehyde, while the right lungs were stored at −80°C for western blotting.

### Cell Culture and Treatment

The alveolar type II epithelial cells of rats (RLE-6TN) were purchased from the American Type Culture Collection (ATCC, United States). RLE-6TN cells were cultured with the RPMI-1640 medium (Hyclone, United States) supplemented with 10% fetal bovine serum (Gibco, United States). RLE-6TN cells were randomly incubated in an atmosphere of 85% O_2_/5% CO_2_ or an atmosphere of 21% O_2_/5% CO_2_ for 48 h. In the NAC treatment experiments, RLE-6TN cells were treated with 10 mM NAC (Sigma-Aldrich, United States). In the Gap26 treatment experiments, we treated RLE-6TN cells with 150 μM Gap26. The drugs and cell culture medium were changed every 24 h.

### Lung Histological and Morphometric Analyses

The fixed lungs were processed to obtain 3 µm thick paraffin sections, which were stained with hematoxylin and eosin (HE) for examination of the lung architecture. Six images were randomly selected for each sample. Alveolarization was evaluated by the radial alveolar count (RAC) value (Cooney and Thurlbeck, 1982) obtained by drawing a line from the center of terminal bronchioles to the nearest connective tissue septum and counting the number of alveoli on the line. ImageJ software was used to measure alveolar wall thickness. These assessments were carried out independently by two pathologists who were blinded to the grouping.

### Immunohistochemical Staining

Paraffin-embedded lung tissue sections were deparaffinized, hydrated, and microwaved in Tris-ethylenediaminetetraacetic acid (EDTA) buffer for antigen retrieval and incubated with 3% hydrogen peroxide to block endogenous peroxidase and then with goat serum to block antibodies. After that, the sections were incubated with primary antibodies (Cx43 rabbit antibody, Bioss, China) overnight at 4°C. The next day, the sections were washed three times with PBS, a secondary antibody was added, and they were incubated at room temperature for 30 min. After adequate washing, the sections were incubated with horseradish enzyme–labeled streptavidin working solution for 20 min at room temperature. Finally, the sections were developed with 3,3′-diaminobenzidine, stained with hematoxylin, dehydrated, and vitrified. The sections were observed under a light microscope.

### TUNEL Staining

Paraffin-embedded lung tissue sections were deparaffinized, hydrated, and washed with PBS three times. Then, the lung tissue sections were treated with the prepared TUNEL test solution (TUNEL kit, Beyotime, China) in a dropwise manner and incubated at room temperature for 60 min. The sections were rewashed with PBS and observed under an optical microscope. Results were evaluated as follows: if the test sample showed strong autofluorescence but the control sample showed no or weak autofluorescence, the result was considered specific positive staining, and positively stained cells were deemed to be apoptotic cells. The apoptosis index (AI) was calculated for each section: AI = number of positive cells/total cells × 100%.

### Apoptotic Cell Rate

In this study, RLE-6TN cells were stained using the Annexin V-FITC/PI apoptosis assay kit (Wanleibio, China) by following the manufacturer’s instructions. After incubation at room temperature for 15 min in the dark, the stained cells were analyzed on a flow cytometer (NovoCyte, Aceabio, United States).

### Western Blotting

Total protein of lung tissues and RLE-6TN cells was obtained with a whole-cell lysis assay (Wanleibio, China). Membrane protein fractions of lung tissue and RLE-6TN cells were obtained with a Mem-PER Plus Membrane Protein Extraction Kit (Thermo Fisher, United States). The samples were loaded onto 4–20% gel, resolved using SDS-PAGE, and subsequently transferred to polyvinylidene fluoride (PVDF) membranes. The PVDF membrane was non-specifically bound to skim milk for 1 h at room temperature and incubated with primary antibodies (Cx43 rabbit antibody, Bioss, China; caspase-3/cleaved caspase-3 rabbit antibody, Wanleibio, China; ASK1 rabbit antibody, Bioss, China; phospho-ASK1 rabbit antibody, Bioss, China; JNK1/2 rabbit antibody, Wanleibio, China; phospho-JNK1/2 rabbit antibody, Wanleibio, China; p38 MAPK rabbit antibody, Wanleibio, China; phospho-p38 MAPK rabbit antibody, Wanleibio, China; Na+/K+-ATPase rabbit antibody, Proteintech, China; β-actin rabbit antibody, Wanleibio, China) overnight at 4°C. The next day, the PVDF membrane was incubated with a secondary antibody (goat anti-rabbit IgG-HRP, Wanleibio, China) at room temperature for 2 h. The proteins were detected by chemiluminescence using an ECL substrate. Finally, digital images were analyzed with ImageJ.

### Real-Time PCR

Lung tissue and RLE-6TN cells were cut in Trizol (9108, Takara, Japan) to isolate and purify the nucleic acid. The A260/A280 ratio was used to adjust the RNA concentration. Primescript RT (Takara, Japan) and SYBR Premier Ex Taq II (Takara, Japan) kits were used for reverse transcription and amplification, respectively. Detection was performed using a Roche LC480 Light Cycler with the following primers: Cx43: forward: 5′-TCT​CGC​CTA​TGT​CTC​CTC​C-3′, reverse: 5′-TGGTC CACGATGGCTAAT-3′; β-actin: forward: 5′-CGT​GCG​TGA​CAT​TAA​AGA​G-3′, reverse: 5′-TTG​CCG​ATA​GTG​ATG​ACC​T-3′. The relative amount of transcripts was calculated using the 2 (−ΔΔCt) method and normalized to β-actin transcript as an internal control.

### ROS Measurement

We centrifuged the homogenates of lung tissues at 1,000 g for 15 min at 4°C and collected the supernatant for subsequent testing. The ROS level was assayed by a ROS assay kit (Wanleibio, China), which used stable non-fluorescent dichlorodihydrofluorescein diacetate (DCFH-DA) as a probe. DCFH-DA freely entered cells and was then hydrolyzed by esterases to create non-fluorescent DCFH. However, DCFH was rapidly oxidized by ROS in the cells to generate strong fluorescent DCF. Hence, ROS levels were assayed indirectly *via* measuring DCF fluorescence.

Next, we detected the ROS level of RLE-6TN cells by flow cytometry analysis using a DCFH-DA fluorescence probe. RLE-6TN cells were trypsinized and collected in 1.5 ml tubes. The cell suspension was then centrifuged at a speed of 2000 rpm for 5 min. After that, the supernatant was removed. The remaining cell pellet was then resuspended in 5 µM DCFH-DA, diluted with culture medium, and continued to incubate for 30 min in the dark. After incubation, the cell pellet was washed twice with PBS and resuspended with 500 µl of PBS. The cell suspension was then subjected to flow cytometry analysis to measure the ROS level on a flow cytometer (NovoCyte, Aceabio, United States).

### Dye-Coupling Assay

GJ function was examined with the dye-coupling assay *in vitro*. Cells were grown to confluence in 12-well plates. Donor cells from one well were incubated with a freshly made solution of 10 μg/ml calcein-AM (Sigma-Aldrich, United States) in a growth medium for 30 min, at 37°C and pH 7.4. Calcein-AM was converted intracellularly into the GJ-permeable dye calcein. Unincorporated dye was removed by three consecutive washes with a culture medium. The donor cells were then trypsinized and seeded onto the receiver cells at the ratio of 1:150 donor/receiver. The cells attached to the monolayer of receiver cells formed GJ channels for 4 h at 37°C and pH 7.4. The results were examined with a fluorescence microscope. The average number of receiver cells containing calcein per donor cell was calculated and considered a measure of the degree of GJ.

### Statistical Analysis

Data analysis was performed using GraphPad Prism version 8.0 (GraphPad software). Experimental data were presented as mean ± SD. Statistical significance between the two groups was determined using Student’s *t*-test. One-way analysis of variance followed by Dunnett’s test was used for multiple group comparisons. *P* < 0.05 was considered statistically significant.

## Results

### Hyperoxia Exposure Resulted in Increased Oxidative Stress and Cx43 Expression *In Vivo* and *In Vitro*

**FIGURE 1:**
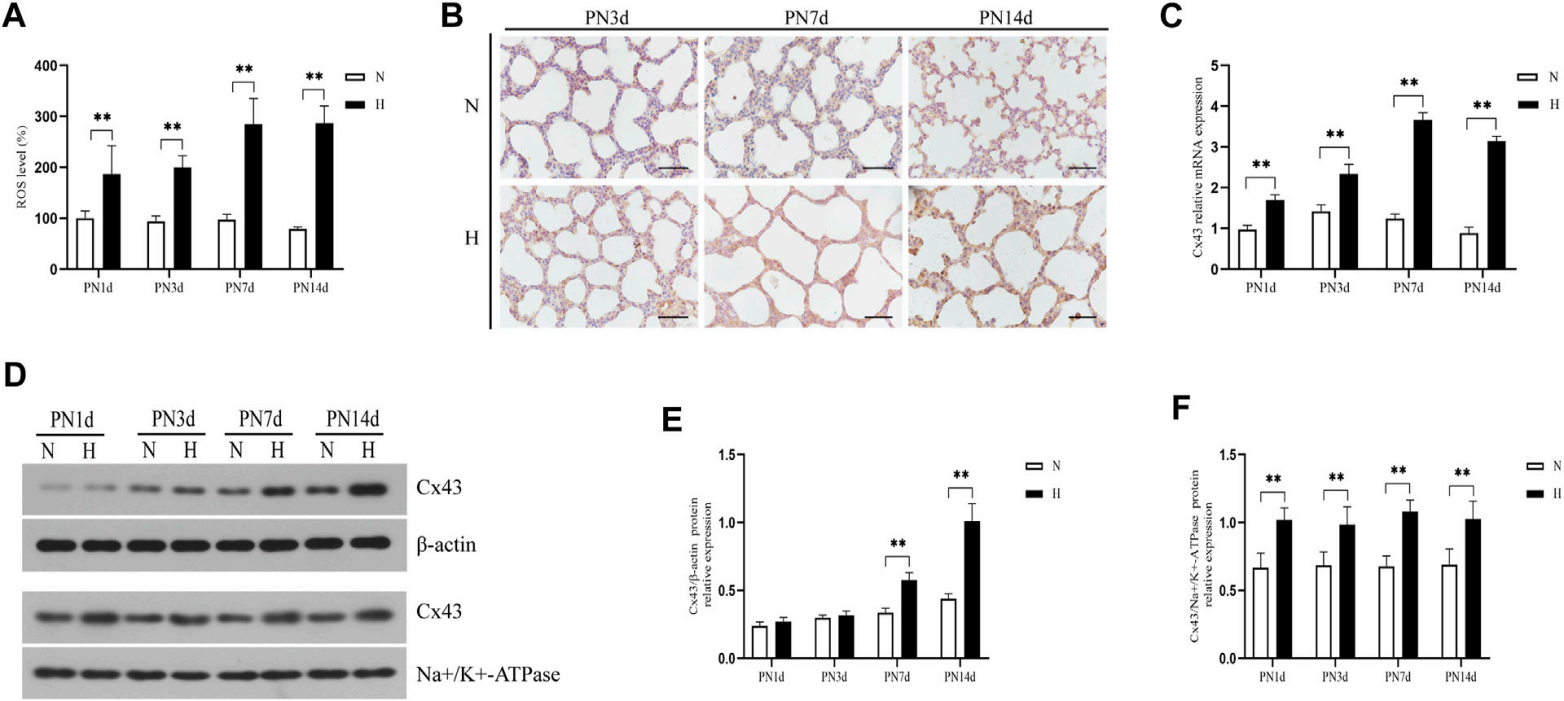
Hyperoxia exposure increased ROS production and Cx43 expression in rats’ lungs. **(A)** The bar chart indicates the ROS level (%). **(B)** Immunohistochemical observation of Cx43 expression (magnification, ×400; scale bar, 50 µm). **(C)** Cx43 gene expression. **(D)** Western blot analyses of Cx43 expression in total and membrane proteins. β-Actin and Na+/K+-ATPase were used as internal references for total and membrane proteins, respectively. **(E)** The bar chart indicates Cx43/β-actin protein relative expression in the total protein. **(F)** The bar chart indicates Cx43/Na+/K+-ATPase protein relative expression in the membrane protein. The data are expressed as mean ± SD from at least six different experiments. **p < 0.01. PN, postnatal day; N, normoxia group; H, hyperoxia group; Cx43, connexin 43; ROS, reactive oxygen species.

To test whether hyperoxia induced an increase in oxidative stress and Cx43 expression *in vivo*, we exposed neonatal rats to 85% O_2_ for 14 days and assessed the ROS level and Cx43 expression in lung tissue. Figure 1A shows that hyperoxia exposure significantly increased ROS production of lung tissue (*p* < 0.01). Cx43 expression in lung tissue was detected by immunohistochemistry, real-time PCR, and western blot. Immunohistochemical staining results showed that Cx43 expression in rats’ lungs was increased by hyperoxia (Figure 1B). Compared with rats exposed to 21% O_2_, Cx43 gene expression in lung tissue of the rats exposed to 85% O_2_ was significantly elevated from PN1d, and this trend stayed till PN14d (Figure 1C, *p* < 0.01). Since GJ channels are located at the cell membrane to mediate intercellular communication, we detected the Cx43 expression in the total and membrane proteins of lung tissues, respectively. Cx43 expression in the total protein of rats’ lungs exposed to 85% O_2_ was significantly higher than that in rats’ lungs exposed to 21% O_2_ at PN7d and PN14d (Figures 1D,E, *p* < 0.01). However, Cx43 expression in the membrane protein was increased in rats’ lungs exposed to hyperoxia from PN1d, and this trend stayed till PN14d (Figures 1D,F, *p* < 0.01). These results suggested that *in vivo*, hyperoxia exposure increased ROS production and increased Cx43 expression.

**FIGURE 2:**
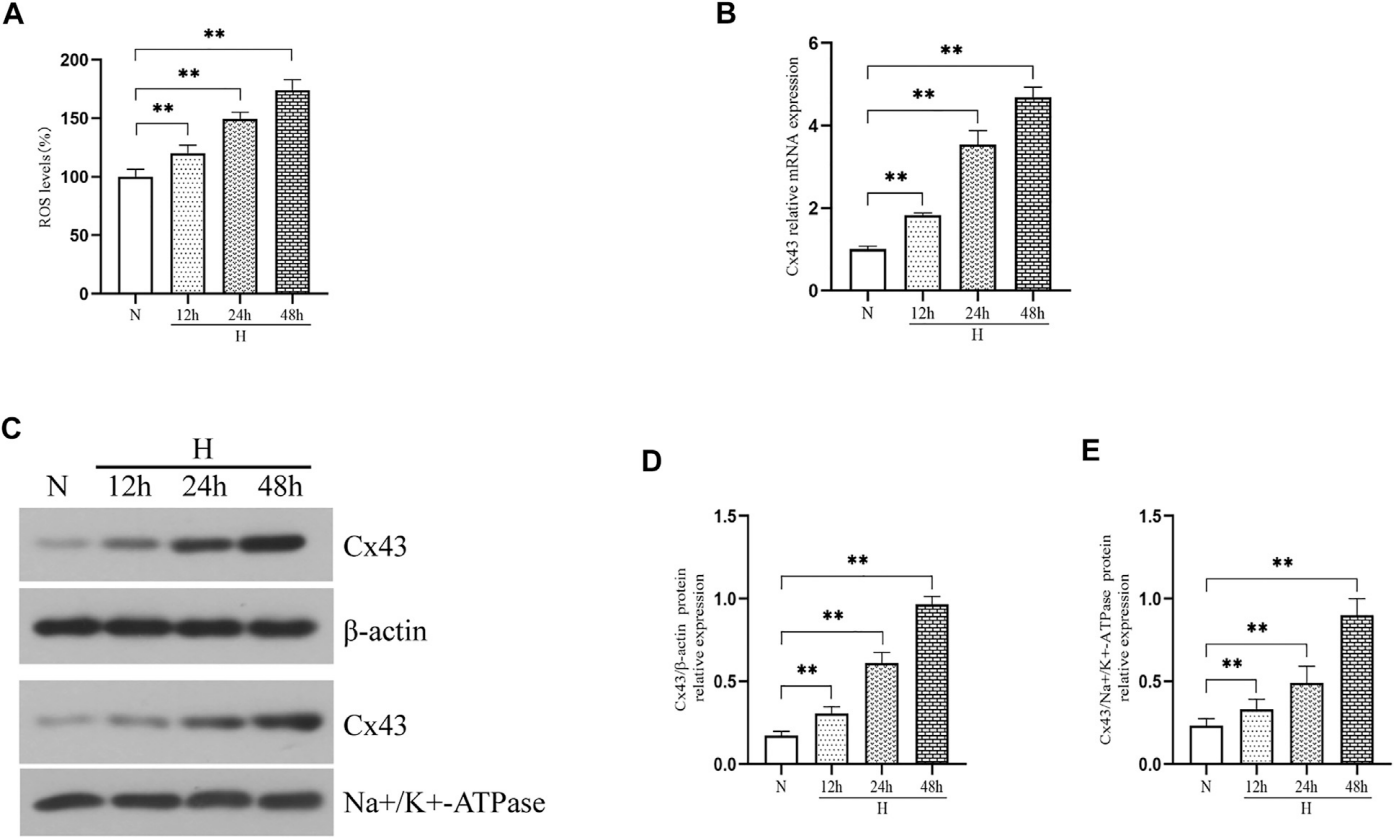
Hyperoxia exposure increased ROS production and Cx43 expression in RLE-6TN cells. **(A)** The bar chart indicates the ROS level (%). **(B)** Cx43 gene expression. **(C)** Western blot analyses of Cx43 expression in total and membrane proteins. β-Actin and Na+/K+-ATPase were used as internal references for total and membrane proteins, respectively. **(D)** The bar chart indicates Cx43/β-actin protein relative expression in the total protein. **(E)** The bar chart indicates Cx43/Na+/K+-ATPase protein relative expression in the membrane protein. The data are expressed as mean ± SD from at least three different experiments. *p < 0.05, **p < 0.01. N, normoxia group; H, hyperoxia group; Cx43, connexin 43; ROS, reactive oxygen species.

To assess whether hyperoxia induced an increase in ROS production and Cx43 expression *in vitro*, we exposed RLE-6TN cells to 85% O_2_ for 48 h and evaluated the ROS level and Cx43 expression. Figure 2A shows that the ROS level in RLE-6TN cells was gradually increased over hyperoxia exposure time (*p* < 0.01). As expected, hyperoxia elevated Cx43 gene and protein expression in RLE-6TN cells (Figures 2B–E, *p* < 0.01).

### Cx43 Expression, Cx43-Mediated Intercellular Communication, and Oxidative Stress Reciprocally Regulated

**FIGURE 3:**
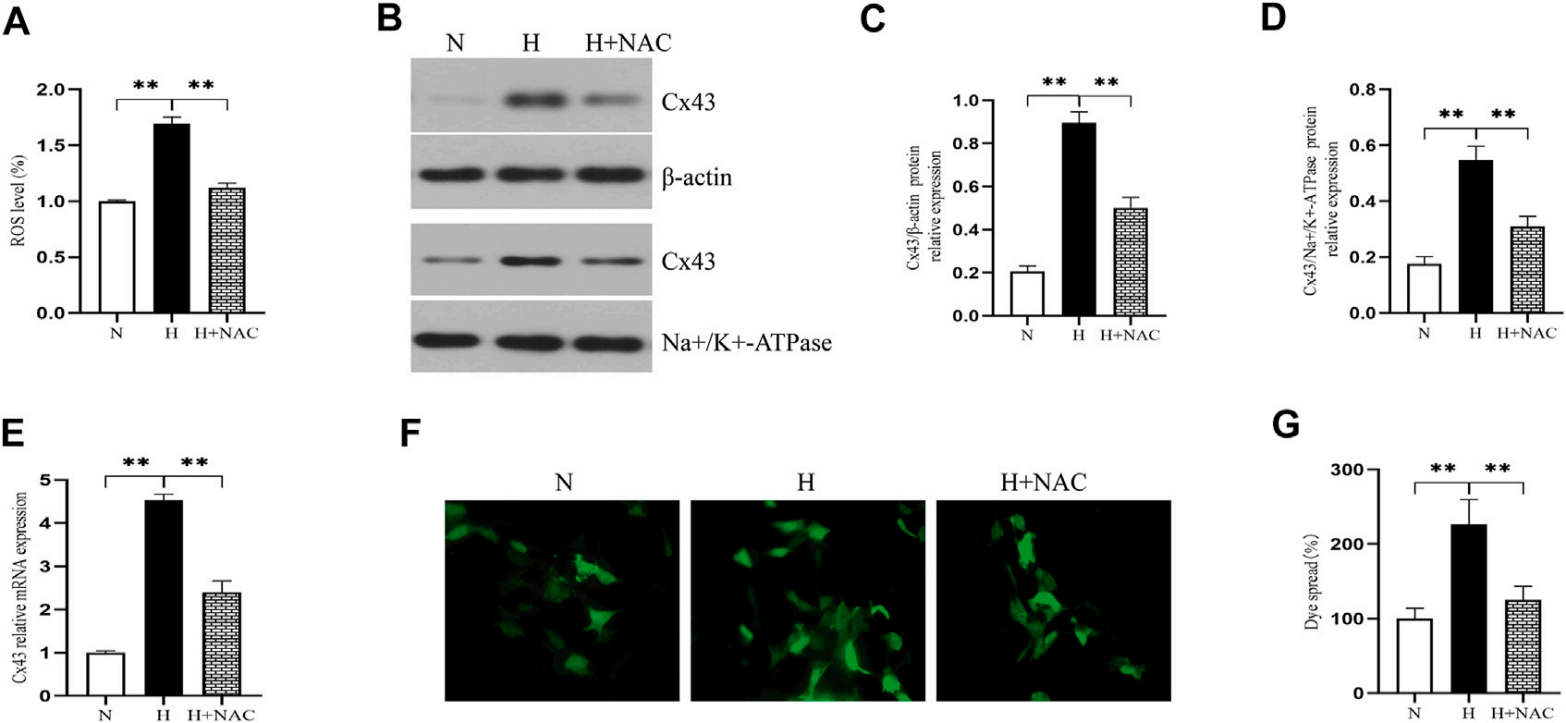
NAC treatment decreased ROS production and Cx43 expression and GJ-mediated intercellular communication. **(A)** The bar chart indicates the ROS level (%). **(B)** Western blot analyses of Cx43 expression in total and membrane proteins. β-Actin and Na+/K+-ATPase were used as internal references for total and membrane proteins, respectively. **(C)** The bar chart indicates Cx43/β-actin protein relative expression in the total protein. **(D)** The bar chart indicates Cx43/Na+/K+-ATPase protein relative expression in the membrane protein. **(E)** Cx43 gene expression. **(F)** Dye-coupling assay. **(G)** The bar chart indicates the diffusion capacity of fluorescent dye between cells (%). The data are expressed as mean ± SD from at least three different experiments. *p < 0.05, **p < 0.01. Cx43, connexin 43; ROS, reactive oxygen species; NAC, N-acetyl cysteine; N, normoxia group; H, hyperoxia group; H+NAC, hyperoxia+NAC (10 mM) group.

We further assessed whether oxidative stress increased Cx43 expression. We exposed RLE-6TN cells to 85% O_2_ for 48 h and treated them with NAC (a kind of ROS scavenger). Figure 3A shows that NAC treatment significantly downregulated the ROS level of RLE-6TN cells in a hyperoxic environment (*p* < 0.01). As expected, NAC treatment decreased the Cx43 gene and protein expression of RLE-6TN cells exposed to hyperoxia (Figures 3B–E, *p* < 0.01). These results indicated that oxidative stress increased Cx43 expression. To assess whether oxidative stress increased GJ-mediated intercellular communication, we subjected RLE-6TN cells to the dye-coupling assay. We found that compared with normoxia exposure, hyperoxia exposure resulted in increased diffusion capacity of fluorescent dye between cells. NAC treatment weakened this capacity, indicating that oxidative stress promoted cell–cell coupling and increased GJ-mediated intercellular communication (Figures 3F–G, *p* < 0.01).

**FIGURE 4:**
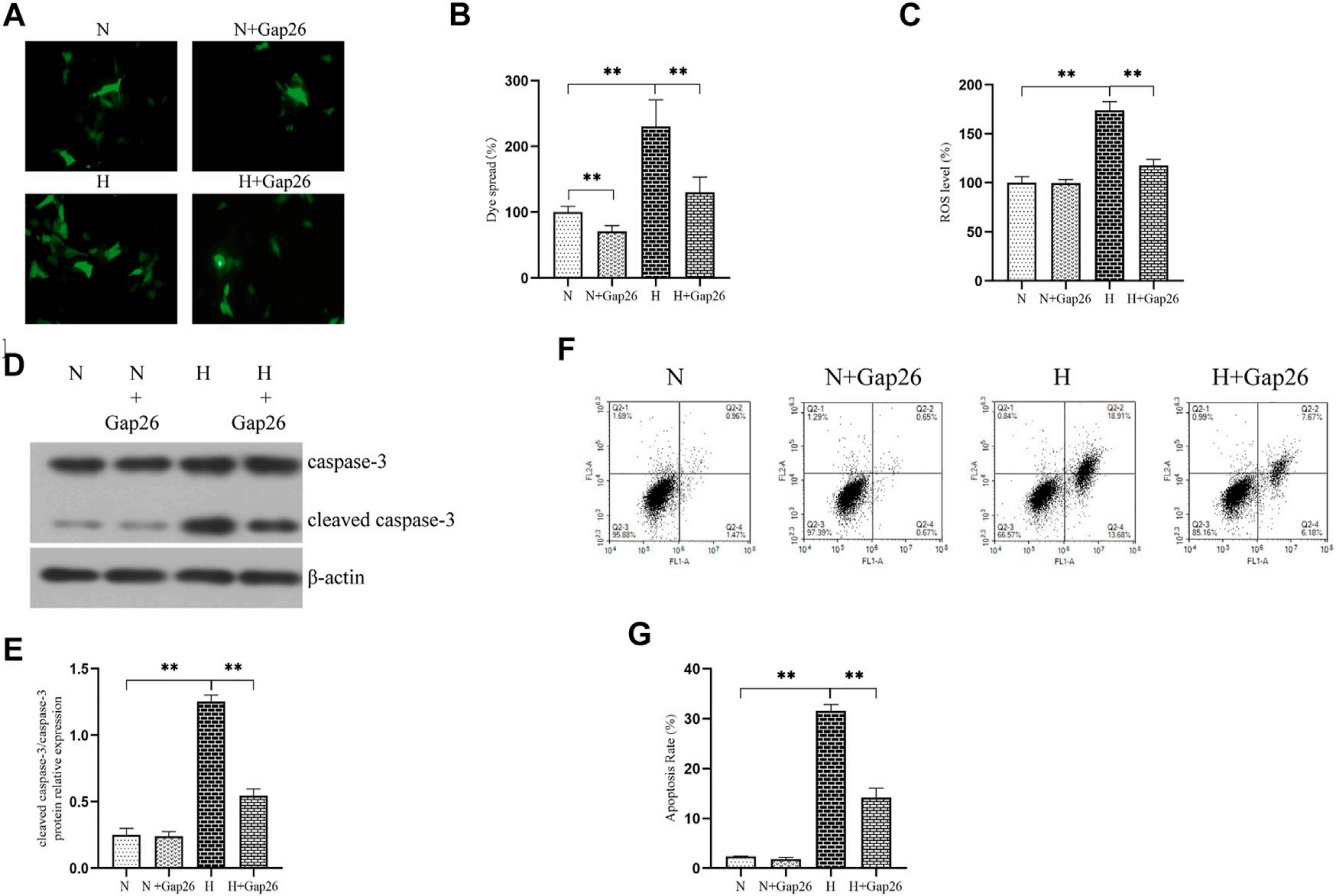
Gap26 treatment decreased ROS production and apoptosis level in RLE-6TN cells exposed to hyperoxia. **(A)** Dye-coupling assay. **(B)** The bar chart indicates the diffusion capacity of fluorescent dye between cells (%). **(C)** The bar chart indicates the ROS level (%). **(D)** Western blot analyses of cleaved caspase-3 and caspase-3 in the total protein. β-Actin was used as the internal reference for the total protein. **(E)** The bar chart indicates the cleaved caspase-3/caspase-3 protein relative expression in the total protein. Protein relative expression was normalized to β-actin expression. **(F)** Flow cytometry using an Annexin V-FITC/PI apoptotic detecting kit. **(G)** The bar chart indicates the apoptosis rate (%). The data are expressed as mean ± SD from at least three different experiments. *p < 0.05, **p < 0.01. Cx43, connexin 43; ROS, reactive oxygen species; N, normoxia group; N+Gap26, normoxia+Gap26 (150 μM) group; H, hyperoxia group; H+Gap26, hyperoxia+Gap26 (150 μM) group.

**FIGURE 5:**
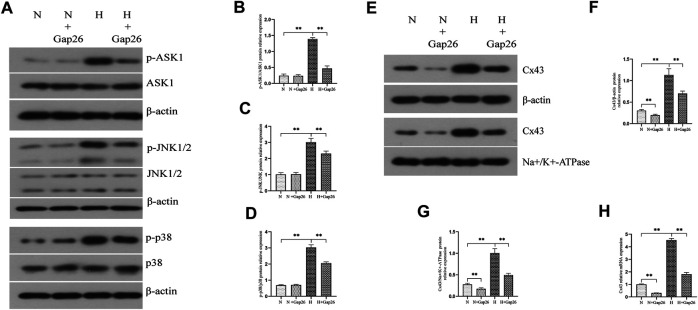
Gap26 treatment inhibited the activation of the ASK1-JNK/p38 signaling pathway and decreased Cx43 expression in RLE-6TN cells exposed to hyperoxia. **(A)** Western blot analyses of p-ASK1, ASK1, p-JNK1/2, JNK1/2, p-p38, and p38 in the total protein. β-Actin was used as the internal reference for the total protein. **(B)** The bar chart indicates p-ASK1/ASK1 protein relative expression in the total protein. Protein relative expression was normalized to β-actin expression. **(C)** The bar chart indicates p-JNK/JNK protein relative expression in the total protein. Protein relative expression was normalized to β-actin expression. **(D)** The bar chart indicates p-p38/p38 protein relative expression in the total protein. Protein relative expression was normalized to β-actin expression. **(E)** Western blot analyses of Cx43 in total and membrane proteins. β-Actin and Na+/K+-ATPase were used as internal references for total and membrane proteins, respectively. **(F)** The bar chart indicates Cx43/β-actin protein relative expression in the total protein. **(G)** The bar chart indicates Cx43/Na+/K+-ATPase protein relative expression in the membrane protein. **(H)** Cx43 gene expression. The data are expressed as mean ± SD from at least three different experiments. *p < 0.05, **p < 0.01. N, normoxia group; N+Gap26, normoxia+Gap26 (150 μM) group; H, hyperoxia group; H+Gap26, hyperoxia+Gap26 (150 μM) group; Cx43, connexin 43; ROS, reactive oxygen species; ASK1, apoptosis signal–regulated kinase 1; JNK, c-Jun NH2-terminal kinase.

We explored whether increased Cx43-GJ–mediated intercellular communication aggravated oxidative stress *in vitro.* We exposed RLE-6TN cells to 85% O_2_ and treated these cells with Gap26. As expected, Gap26 treatment significantly weakened the GJ-mediated intercellular communication (Figures 4A,B, *p* < 0.01). Figure 4C shows that Gap26 decreased ROS production (*p* < 0.01) of RLE-6TN in hyperoxia exposure. Moreover, we detected the effect of Gap26 on the ASK1-JNK/p38 signaling pathway and apoptosis. We found that Gap26 decreased the cleaved caspase-3/caspase-3 ratio (Figures 4D,E, *p* < 0.01) and apoptotic rate of RLE-6TN cells in hyperoxia exposure (Figures 4F,G, *p* < 0.01). As demonstrated in Figure 5A and quantified in Figures 5B–D, RLE-6TN cells exposed to hyperoxia had increased phosphorylated protein expression of ASK1, JNK1/2, and p38 MAPK, while Gap26 treatment significantly reversed these changes. Based on the above results, we believed that Gap26 treatment alleviated oxidative stress, thus decreasing apoptosis *via* inhibiting ASK1-JNK/p38 signaling *in vitro*. Of note, we found Gap26 also inhibited the expression of Cx43 (Figures 5E–H, *p* < 0.01). We supported that ROS increased Cx43 expression, and Gap26 treatment inhibited the effect *via* decreasing ROS production.

### Gap26 Improved Alveolar Development in Neonatal Rats With Hyperoxia Exposure

**FIGURE 6:**
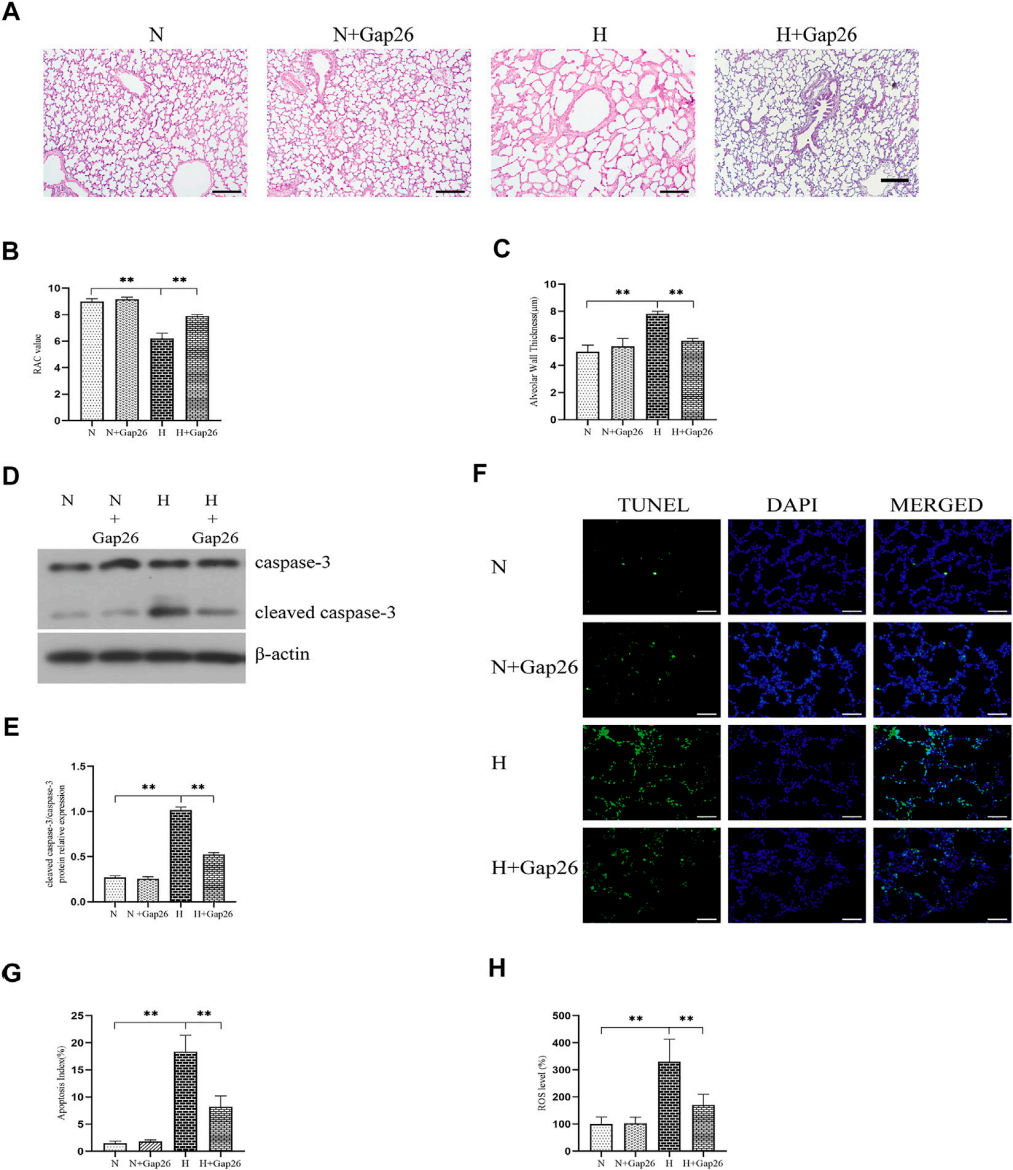
Gap26 treatment improved alveolar development, decreased oxidative stress, and decreased apoptosis of rats’ lungs exposed to hyperoxia. **(A)** HE staining of lung tissue sections (magnification, ×100; scale bar, 200 µm). Changes in lung morphology were quantified using the **(B)** RAC value and **(C)** alveolar wall thickness. **(D)** Western blot analyses of cleaved caspase-3 and caspase-3 in the total protein. β-Actin was used as the internal reference for the total protein. **(E)** The bar chart indicates the cleaved caspase-3/caspase-3 protein relative expression in the total protein. Protein relative expression was normalized to β-actin expression. **(F)** TUNEL staining of lung tissues (magnification, ×400; scale bar, 50 µm). **(G)** The bar chart indicates the apoptosis index (%). **(H)** The bar chart indicates the ROS level (%) in lung tissue. The data are expressed as mean ± SD from at least six different experiments. *p < 0.05, **p < 0.01. RAC, radial alveolar count; Cx43, connexin 43; ROS, reactive oxygen species; N, normoxia+saline group; N+Gap26, normoxia+Gap26 (50 ug/kg/d) group; H, hyperoxia+saline group; H+Gap26, hyperoxia+Gap26 (50 ug/kg/d) group.

**FIGURE 7:**
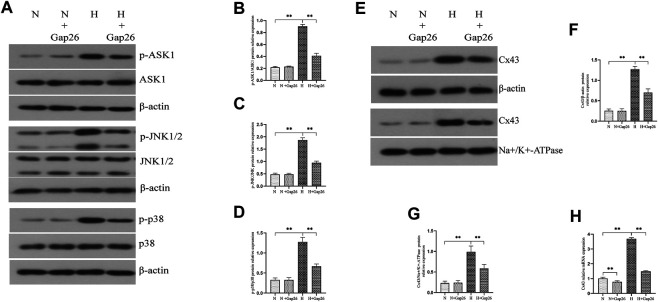
Gap26 treatment inhibited the activation of the ASK1-JNK/p38 signaling pathway and decreased Cx43 expression of rats’ lungs exposed to hyperoxia. **(A)** Western blot analyses of p-ASK1, ASK1, p-JNK1/2, JNK1/2, p-p38, and p38 in the total protein. β-Actin was used as an internal reference for the total protein. **(B)** The bar chart indicates p-ASK1/ASK1 protein relative expression in the total protein. Protein relative expression was normalized to β-actin expression. **(C)** The bar chart indicates p-JNK/JNK protein relative expression in the total protein. Protein relative expression was normalized to β-actin expression. **(D)** The bar chart indicates p-p38/p38 protein relative expression in the total protein. Protein relative expression was normalized to β-actin expression. **(E)** Western blot analyses of Cx43 in total and membrane proteins. β-Actin and Na+/K+-ATPase were used as the internal references for total and membrane proteins, respectively. **(F)** The bar chart indicates Cx43/β-actin protein relative expression in the total protein. **(G)** The bar chart indicates Cx43/Na+/K+-ATPase protein relative expression in the membrane protein. **(H)** Cx43 gene expression. The data are expressed as mean ± SD from at least six different experiments. *p < 0.05, **p < 0.01. Cx43, connexin 43; ROS, reactive oxygen species; ASK1, apoptosis signal–regulated kinase 1; JNK, c-Jun NH2-terminal kinase; N, normoxia+saline group; N+Gap26, normoxia+Gap26 (50 ug/kg/d) group; H, hyperoxia+saline group; H+Gap26, hyperoxia+Gap26 (50 ug/kg/d) group.

To test whether Gap26 had a protective effect against hyperoxia-induced BPD, we exposed the neonatal rats to 85% O_2_ and treated them with Gap26 continuously from postnatal day (PN) 1 to 14. HE staining results (Figure 6A) showed that the hyperoxia-exposed and Gap26-treated rats had increased alveolarization with a higher RAC value (Figure 6B, *p* < 0.01) and thinner alveolar wall thickness (Figure 6C, *p* < 0.01) compared to the rats with hyperoxia exposure alone. These results demonstrated that Gap26 treatment improved the alveolar development of neonatal rats with hyperoxia exposure. As shown in Figure 6D and quantified in Figure 6E, treatment with Gap26 reversed the increase in the cleaved caspase-3/caspase-3 ratio under hyperoxia conditions (*p* < 0.01). TUNEL staining results showed that compared to the rats with hyperoxia exposure alone, hyperoxia-exposed and Gap26-treated rats had lower fluorescence intensity (Figure 6F). Gap26 treatment decreased the apoptosis index of rats exposed to hyperoxia (Figure 6G, *p* < 0.01). Figure 6H shows that Gap26 decreased ROS production of rats’ lungs with hyperoxia exposure (*p* < 0.01). Moreover, we found that Gap26 treatment reduced the activity of the ASK1-JNK/p38 signaling pathway in rats exposed to hyperoxia (Figures 7A–D, *p* < 0.01). Consistent with the *in vitro* experiment, Gap26 treatment also decreased the Cx43 gene and protein expression of rats’ lungs under a hyperoxic environment (Figures 7E–H, *p* < 0.01).

## Discussion

In this study, the rat model of BPD was developed by exposing neonatal Sprague Dawley rats to a hyperoxic environment. Firstly, we found that ROS production and Cx43 expression were significantly increased in BPD rats. Next, to explore the relationship between Cx43 and oxidative stress, type II alveolar epithelial cells of rats were cultured in a hyperoxic environment and treated with NAC (a kind of ROS scavenger) or Gap26 (a specific Cx43-GJ blocker). We found that ROS increased Cx43 expression and Cx43-GJ–mediated intercellular communication; Gap26 treatment downregulated ROS production, inhibited the ASK1-JNK/p38 signaling pathway, and decreased apoptosis. These results indicated that oxidative stress increased Cx43 expression and Cx43-GJ–mediated intercellular communication; moreover, Cx43-GJ aggravated cell apoptosis induced by oxidative stress *via* the ASK1-JNK/p38 signaling pathway. Finally, we treated neonatal rats in a hyperoxic environment with Gap26. Our results indicated that Gap26 treatment improved alveolar development in neonatal rats with hyperoxia exposure. According to the above results, this study demonstrates that Cx43 is involved in alveolar maldevelopment of hyperoxia-induced BPD. The specific connexin 43–inhibiting peptide Gap26 was a novel therapeutic strategy of BPD.

BPD has become the most common complication in premature infants (Siffel et al., 2021). Oxidative stress is one of the vital pathogeneses of BPD. Newborns pass from the womb into a relatively hyperoxic environment. They often experience increased oxidative stress and have elevated reactive oxygen species (ROS) production. Premature infants with immature antioxidant defense systems are more susceptible to lung injury induced by oxidative stress, and either oxygen treatment or mechanical ventilation may cause further lung injury by oxidative stress and interrupted pulmonary alveolar and vascular development, eventually contributing to BPD (Kinsella et al., 2006). Alveolar epithelial cells are very susceptible to oxidative stress injury. Type II alveolar epithelial cells (ATII cells), as the pulmonary progenitor cells, can self-renew or transdifferentiate into type I alveolar epithelial cells (ATI cells). However, under sustained oxidative stress, ATII cells lose the ability of self-renewal and *trans*-differentiation and even trigger cell death. The excessive apoptosis of alveolar epithelial cells induced by oxidative stress is the crucial mechanism in the alveolar maldevelopment of BPD. In this study, our results showed that hyperoxia exposure increased oxidative stress and elevated apoptotic levels *in vivo* and *in vitro*. A large number of studies have demonstrated that the ASK-JNK/p38 signaling pathway is activated by oxidative stress and induced apoptosis (Tobiume et al., 2001; Li et al., 2003; Romashko et al., 2003; Zhang et al., 2003; Kadowaki et al., 2005; Watanabe et al., 2005; Huang et al., 2009). In this study, we detected the activation of the ASK-JNK/p38 signaling pathway. As expected, hyperoxia exposure activated the ASK-JNK/p38 signaling pathway *in vivo* and *in vitro*. In general, we demonstrated that hyperoxia exposure increased oxidative stress, activating the ASK-JNK/p38 signaling pathway and causing excessive apoptosis *in vivo* and *in vitro*.

Previous studies have indicated that GJ is involved in various critical physiological processes such as cell homeostasis, proliferation and differentiation, and apoptosis (Kumar and Gilula, 1996). Recently, increased intercellular communication by GJ primarily composed of Cx43 was implicated in several pathogeneses, such as chronic inflammation, DNA damage, ER stress, and oxidative stress (Matus et al., 2011; Zou et al., 2019; Hoorelbeke et al., 2020; Tirosh et al., 2021). These observations indicate that increased intercellular communication may be a common feature of conditions characterized under cellular stress. The increased intercellular communication may be deleterious to tissue homeostasis in the setting of cellular stresses. For example, in hepatocytes of obese mice, Cx43 expression is upregulated under ER stress conditions, leading to increased Cx43-mediated intercellular communication. The increased cell–cell coupling allows transmission of ER stress signals from stressed to neighboring, ER-stress–naive bystander cells, resulting in impaired ER function and insulin resistance (Tirosh et al., 2021). In this study, we demonstrated that ROS increased Cx43 expression and Cx43-mediated intercellular communication. Also, our results indicated that *in vitro*, blocking Cx43-mediated intercellular communication by Gap26 treatment could decrease ROS production, thus inhibiting the ASK1-JNK/p38 signaling pathway and downregulating the apoptotic level. Therefore, our results supported that oxidative stress increased Cx43 expression and Cx43-mediated intercellular communication, which could further amplify oxidative stress–mediated apoptosis. And Gap26 could inhibit the amplification of GJ to oxidative stress. Therefore, we believed that there is a reciprocal modulation between Cx43 expression and Cx43-mediated intercellular communication and oxidative stress. Interestingly, in this study, we found that Gap26 treatment, as a GJ functional inhibitor, also decreased Cx43 expression. The possible mechanism is that ROS increased Cx43 expression and Gap26 treatment inhibited the effect *via* reducing ROS production.

Gap26 has been used in some disease models *in vivo*, such as ischemic brain injury (Li et al., 2015), myocardial infarction (Hawat et al., 2012), and pulmonary diseases (Huang et al., 2018). In this study, we treated neonatal rats in 85% O_2_ with Gap26. We found that Gap26 treatment decreased the ROS level, inhibiting the ASK1-JNK/p38 signaling pathway and downregulating the apoptotic level of lung tissues, and finally improved the alveolar development of BPD rats. Our results indicated that Gap26 is protective against oxidative stress–mediated apoptosis and alveolar maldevelopment of BPD rats.

However, Gap26 treatment in this study has potential limitations. Gap26 is developed as a Cx43 mimetic peptide, aiming to interfere with Cx43 channel function. Although an increasing number of studies report on the essential effects of this compound on disease models, the specific actions of the mimetic peptide are not yet fully understood, and diverse questions remain, including its stability, administration mode, and adverse effects. Therefore, further experiments about the stability, administration modes, and side effects’ observation of Gap26 are needed.

## Conclusion

Cx43 and Cx43-mediated intercellular communication were involved in alveolar maldevelopment of BPD. Cx43 expression, Cx43-mediated intercellular communication, and oxidative stress are reciprocally regulated. The specific Cx43-inhibiting peptide Gap26 was a novel therapeutic strategy of BPD.

## Data Availability

The raw data supporting the conclusions of this article will be made available by the authors, without undue reservation, to any qualified researcher.
